# Wing geometric morphometrics to distinguish and identify *Haematobosca* flies (Diptera: Muscidae) from Thailand

**DOI:** 10.1016/j.ijppaw.2023.04.008

**Published:** 2023-04-20

**Authors:** Nusara Ardkhongharn, Romyakorn Ravichotikul, Patthanan Aksornchai, Thekhawet Weluwanarak, Tanawat Chaiphongpachara, Tanasak Changbunjong

**Affiliations:** a Department of Pre-Clinic and Applied Animal Science, Faculty of Veterinary Science, Mahidol University, Nakhon Pathom, 73170, Thailand; b The Monitoring and Surveillance Center for Zoonotic Diseases in Wildlife and Exotic Animals (MoZWE), Faculty of Veterinary Science, Mahidol University, Nakhon Pathom, 73170, Thailand; c Department of Public Health and Health Promotion, College of Allied Health Sciences, Suan Sunandha Rajabhat University, Samut Songkhram, 75000, Thailand

**Keywords:** Landmark, Morphometry, *Haematobosca aberrans*, *Haematobosca sanguinolenta*, Stomoxyinae, Vector

## Abstract

The hematophagous flies of the genus *Haematobosca* Bezzi, 1907 (Diptera: Muscidae) are important ectoparasites in domestic animals and wildlife. Two species of this genus have been recorded in Thailand, viz., *Haematobosca sanguinolenta* (Austen, 1909) and *Haematobosca aberrans* (Pont, Duvallet & Changbunjong, 2020). They have a similar morphology and coexist in the same habitat. The correct species identification of these flies is extremely important for understanding disease epidemiology and developing effective control measures. Geometric morphometrics (GM) has been confirmed to be a useful tool for differentiating and identifying morphologically similar insect species. Therefore, GM was used to distinguish and identify *H. sanguinolenta* and *H. aberrans* in Thailand*.* Adult flies of both sexes were collected using Nzi traps, morphologically identified, and analyzed by landmark-based GM of the wing. Results showed that GM was highly effective in distinguishing the two *Haematobosca* species based on their wing shape, with an overall accuracy score of 99.3%. We also revealed that our study material could be used as reference data to identify new field specimens collected from other geographic locations. We propose that wing GM can be used as a supplement to conventional morphology identification, particularly for *Haematobosca* specimen that has been damaged or has lost its diagnostic characteristics due to specimen collection and processing in the field.

## Highlights

• *Haematobosca sanguinolenta* and *H. aberrans* are morphologically similar.
• Wing geometric morphometrics is effective in distinguishing between two *Haematoboca* species.
• Geometric morphometric data can be used as a reference to identify new field specimens.

## Graphical abstract

**Figure:**
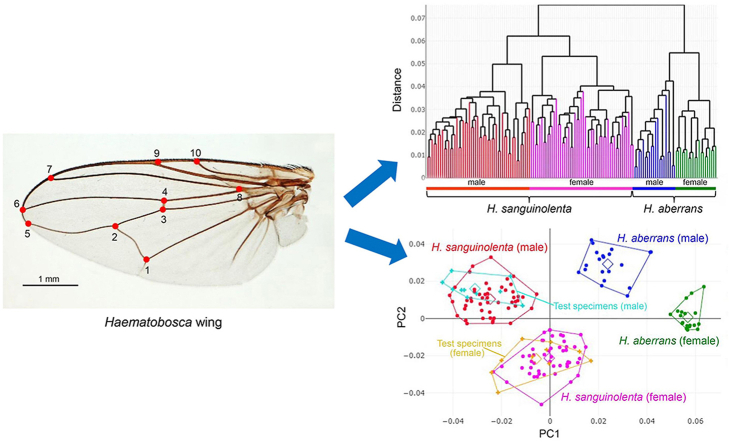

## 1 Introduction

The genus *Haematobosca* Bezzi, 1907 (Diptera: Muscidae) consists of bloodsucking flies belonging to the subfamily Stomoxyinae that contains 16 described species (Braack and Pont, 2012; Pont et al., 2020). Most species are distributed in the Afrotropical region, and other species are limited to the Palaearctic, Holarctic, Oriental, and Australasian regions (Braack and Pont, 2012; Pont et al., 2020). All *Haematobosca* species are important ectoparasites of cattle, horses, and wild ungulates (Zumpt, 1973; Garros et al., 2018). In Thailand, two species of *Haematobosca*, including *Haematobosca sanguinolenta* (Austen, 1909) and *Haematobosca aberrans* (Pont, Duvallet & Changbunjong, 2020), have been recorded (Zumpt, 1973, Tumrasvin and Shinonaga, 1978; Changbunjong et al., 2012; Changbunjong et al., 2013; Pont et al., 2020). Among these two species, it was discovered that *H. sanguinolenta* was the most abundant in the national park, followed by the livestock farm and wildlife conservation area (Changbunjong et al., 2012). This species was suspected as an important vector of *Theileria cervi* in sambar deer at the Khoa Yai National Park (Changbunjong et al., 2016b). It is known that the parasite can cause asymptomatic and severe forms and also induce death in cervids (Pavón-Rocha et al., 2020). In contrast, *H. aberrans*, recently reported as a new species, was found in a livestock farm near a forest area (Pont et al., 2020).

**Fig. 1:**
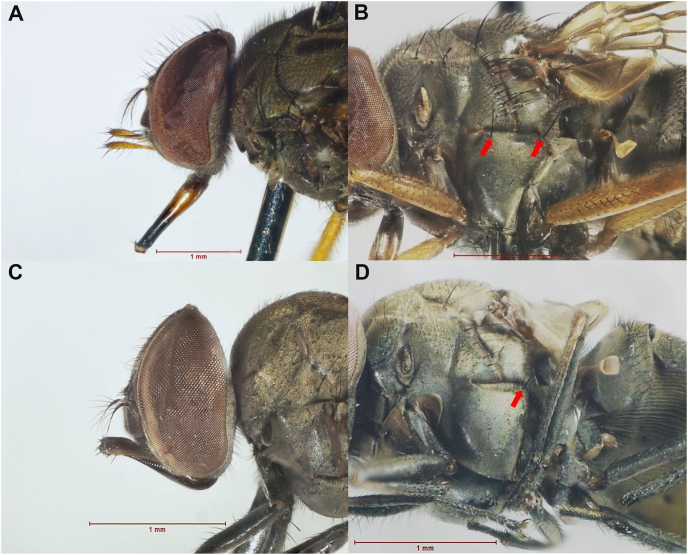
Heads in the lateral view and the pleura of *Haematobosca sanguinolenta* (**A**, **B**) and *H. aberrans* (**C**, **D**). The anterior and posterior katepisternal setae (arrow) were used to distinguish between both species. Photographs were prepared by the authors.

The flies of the genus *Haematobosca* can be distinguished from the other stomoxyine flies (*Bruceomyia*, *Haematobia*, *Haematostoma*, *Neivamyia*, *Parastomoxys*, *Prostomoxys*, *Stomoxys*, and *Stygeromyia*) based on the presence of dorsal and ventral hairs on their arista. In addition, the palpi are internally grooved and of the same length as the proboscis, and the katepisternum has anterior and posterior katepisternal setae (also known as sternopleural bristles), with the exception of *H. aberrans* in which the anterior katepisternal seta is absent (Zumpt, 1973; Pont et al., 2020). Consequently, the presence of an anterior katepisternal seta can be used to differentiate between *H. sanguinolenta* and *H. aberrans* that are found in Thailand (Fig. 1). Despite the fact that these species can be easily distinguished by this characteristic, *H. sanguinolenta* might resemble *H. aberrans* if an anterior katepisternal seta is lost during specimen collection and processing in the field.

The correct species identification of insect vectors is extremely important for understanding disease epidemiology and developing effective control strategies. Therefore, to resolve the problems from the conventional morphological identification, alternative methods such as molecular identification and geometric morphometrics (GM) are now extremely useful (Dujardin, 2008; Changbunjong et al., 2016c, 2020). The molecular-based identification method, namely, DNA barcoding, has been confirmed to be effective for the identification of *H. sanguinolenta* and *H. aberrans* (Changbunjong et al., 2020). This method uses a ∼650-bp fragment of the mitochondrial cytochrome oxidase subunit I (*COI*) gene as the DNA barcode for species identification (Hebert and Gregory, 2005; Changbunjong et al., 2020). Although the molecular method can provide 100% accuracy in the species identification of *Haematobosca* flies, it is time-consuming, and requires expensive equipment and laboratory expertise. In contrast, GM is a rapid, easy, and affordable method that requires minimal expensive equipment or laboratory expertise. It is a quantitative method for measuring the size and shape variation through landmark-, semilandmark-, and outline-based analyses (Dujardin 2008; Dujardin et al., 2014; Dujardin and Dujardin, 2019). The GM method can be used to identify species and evaluate differences within species (intraspecific variation) and between sexes (sexual dimorphism) in several insects of medical and veterinary importance (Ruangsittichai et al., 2011; Lorenz et al., 2012; Sontigun et al., 2017; De Souza et al., 2020; Oliveira-Christe et al., 2020; Simões et al., 2020; Changbunjong et al., 2021; Martinet et al., 2021; Chaiphongpachara et al., 2022a; Chaiphongpachara et al. 2022b; Chaiphongpachara et al. 2022c; Laojun et al., 2023). The efficiency of GM for species identification has been demonstrated in stomoxyine flies (*Stomoxys* spp.) (Changbunjong et al., 2016a, 2023) and other Dipterans such as blow flies (Sontigun et al., 2017), horse flies (Changbunjong et al., 2021), mosquitoes (Lorenz et al., 2012; De Souza et al., 2020; Simões et al., 2020; Martinet et al., 2021; Chaiphongpachara et al., 2022a; Laojun et al., 2023), sand flies (Dujardin et al., 2003; Giordani et al., 2017), and tsetse flies (Kaba et al., 2017). Therefore, in this study, the landmark-based GM of the wing was used to distinguish and identify *H. sanguinolenta* and *H. aberrans* in Thailand*.* We propose the wing GM as a new alternative method to help the morphological identification of *Haematobosca* species.

## 2 Materials and methods

### 2.1 Ethical statement

All procedures used in this study were approved by the Faculty of Veterinary Science, Mahidol University Animal Care, and Use Committee, with the ethics approval reference MUVS-2021-11-50.

### 2.2 Specimen collection and species determination

**Fig. 2:**
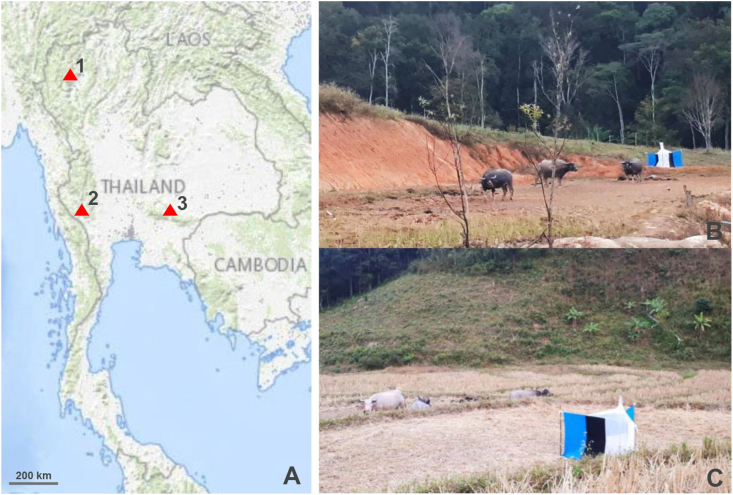
The topographic map of the *Haematobosca* fly collection sites in Thailand: Chiang Mai (1), Kanchanaburi (2), and Nakhon Ratchasima (3) (A). The Nzi trap used for fly collection was placed near animal hosts at each collection site (B, C). This map was prepared from the United States Geological Survey (USGS) National Map Viewer available at http://viewer.nationalmap.gov/viewer/, accessed on February 10, 2023.

*H. sanguinolenta* and *H. aberrans* flies of both sexes were collected from a buffalo farm close to a forest in Chiang Mai Province, northern Thailand (Table 1, Fig. 2). Five Nzi traps (Mihok, 2002) were positioned near the animal hosts for 4 days from 06:00 to 18:00 in February 2022. The specimens were collected from the traps at 2- or 3-h intervals to avoid specimen damage, and then they were immediately euthanized by freezing at −10 °C. All specimens were individually placed in 1.5-mL microcentrifuge tubes for secure storage before being transported to the Vector-Borne Diseases Research Unit, Faculty of Veterinary Science, Mahidol University, Nakhon Pathom Province, central Thailand.

**Table 1:** Collection sites and number (*n*) of specimens of *Haematobosca* flies used for the wing geometric morphometric analysis.

Species	Province (Coordinate)	Region	Month/Year	*n*
*H. sanguinolenta*	Chiang Mai (N18°38′35″, E98°31′27″)	Northern	February 2022	Male 50, Female 50
	Kanchanaburi^a^ (N14°25′53″, E98°48′35″)	Western	March 2022	Male 5, Female 5
	Nakhon Ratchasima^a^ (N14°22′23″, E101°44′51″)	Northeastern	November 2022	Male 5, Female 5
*H. aberrans*	Chiang Mai (N18°38′35″, E98°31′27″)	Northern	February 2022	Male 20, Female 20

a , test specimens.

Species determination was conducted from the specimens with distinct morphological diagnostic characteristics using the taxonomic keys described by Zumpt (1973) and Pont et al. (2020) under a stereomicroscope (Nikon SMZ745; Nikon Corp., Tokyo, Japan).

To assess the reliability of the morphometric analysis based on 140 reference wings to differentiate species, additional specimens (also known as test specimens) were collected from other geographic locations. They comprised *H. sanguinolenta* from Kanchanaburi and Nakhon Ratchasima Provinces, Thailand (Table 1, Fig. 2), which were divided into males (n = 10) and females (n = 10). All of them had clear morphological diagnostic characteristics.

### 2.3 Wing GM analysis

#### 2.3.1 Wing preparation and landmark digitization

**Fig. 3:**
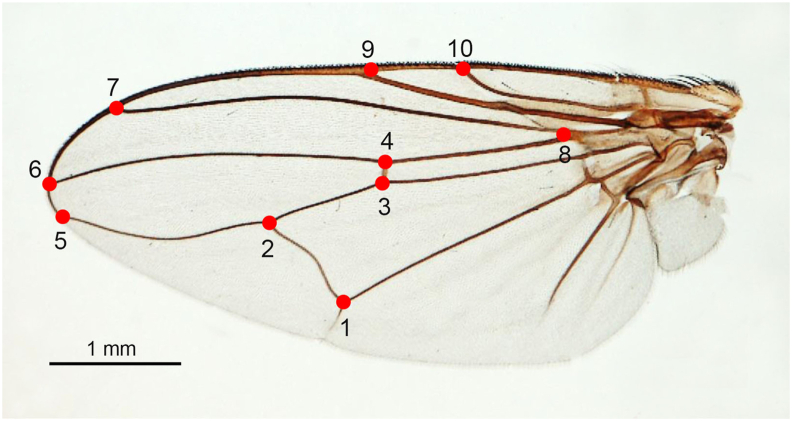
The 10 landmarks on the wing of *Haematobosca* flies used in the wing geometric morphometric analysis.

The left wing of each male and female *H. sanguinolenta* and *H. aberrans* was detached from the thorax using a sterile blade and mounted between a microscope slide and a cover glass with Hoyer's medium (Changbunjong et al., 2020). All the mounted wing slides were photographed using a digital camera linked to a stereomicroscope (Nikon AZ 100; Nikon Corp., Tokyo, Japan) at 10x magnification, and a scale bar of 1 mm was placed on wing images to avoid wing sizing errors. For GM analysis, 10 wing landmarks (Fig. 3) were digitized based on a previous study by Changbunjong et al. (2020) (Table S1).

#### 2.3.2 Repeatability

A repeatability test for shape was conducted to evaluate the accuracy of landmark digitization, and the result was expressed as a repeatability index. It was computed based on the Procrustes analysis of variance (ANOVA) method (Klingenberg and McIntyre, 1998). A total of 10 wing images of each species were randomly chosen and digitized twice by the same user (for intra-user repeatability) and different users (for inter-user repeatability). In this study, all wing images were re-digitized if the intra- and inter-user repeatability values were < 90% (Changbunjong et al., 2021, 2023).

#### 2.3.3 Wing size analysis

The centroid size (CS), which is derived from the coordinates of all landmarks, was used to estimate the global wing size to demonstrate the size variation in male and female *H. sanguinolenta* and *H. aberrans* flies using a graphic boxplot. The wing CS is determined by the square root of the total of squared distances measured from the centroid to each of the landmarks (Bookstein, 1991). The one-way ANOVA was used to compare the means of the CS among groups for each species and sex. A nonparametric permutation test (1000 permutations) with a Bonferroni correction was performed to determine the statistical significance of the one-way ANOVA at a *p* value of 0.05.

#### **2.3.4** Wing shape analysis

The wing shape variables, also known as orthogonal projections, were obtained after Procrustes superimposition using the generalized Procrustes analysis (GPA) (Rohlf, 1990). The principal components (PCs) of wing shape variables were used as final shape variables for wing shape analysis. The superimposition of all configurations resulted in a visual comparison of the mean shape (consensus) between species and sexes. Principal component analysis (PCA) of wing shape variables was performed to visualize the morphospace of individuals, which was illustrated by the factor map of the first two PCs. The discriminant analysis used to separate species as well as sexes was performed using the final wing shape variables, as shown by the factor map of the first two discriminant factors (DFs). A nonparametric permutation test (1000 permutations) with a Bonferroni correction was used to determine the statistical significance of the Mahalanobis distances between species and sexes at a *p* value of 0.05.

Unsupervised classification using an unweighted pair group method with arithmetic mean based on the Euclidean distances between shape variables was used to generate the hierarchical agglomerative clustering (HAC) tree (Rajonhson et al., 2022), which displays the pattern of the similarities and differences of wing shape between individuals.

#### 2.3.5 Validated classification

A cross-validated classification was used to calculate the percentage of specimens that were accurately identified as belonging to their respective species and sexes. According to the maximum likelihood method (Dujardin et al., 2017) and Mahalanobis distance (Manly, 2004), each individual sample was successively removed from the total sample and assigned to the most likely group for wing size and the closest group for wing shape, respectively.

#### 2.3.6 Allometry

The allometry, or the effect of wing size on wing shape variation, was analyzed using linear regression between the wing CS and the first PC. The allometric effect was then evaluated using the determination coefficient (r^2^).

#### 2.3.7 Identification of test specimens

Our study material (the raw coordinates of wing landmarks) was used as reference data to determine 20 test specimens. The test specimens collected from other geographic locations were used as specimens to be identified using the following four reference groups in our study: 1) male *H. sanguinolenta*, 2) female *H. sanguinolenta*, 3) male *H. aberrans*, and 4) female *H. aberrans*.

The identification method was performed individually for the test specimens. To classify test specimens as members of a specific species, the PCA was performed in the same manner as performed for wing shape analysis, where the morphospace of reference data was used for each test specimen. In addition, an HAC tree based on the Euclidean distances between shape variables was used to illustrate the similarity between test specimens and reference data.

#### 2.3.8 Morphometric software

The XYOM (XY Online Morphometrics) version 2 was used to digitize landmarks, generate graphical output, and conduct statistical analysis (Dujardin and Dujardin, 2019). This morphometric software is freely accessible at https://xyom.io/, accessed on January 30, 2023.

## 3 Results

### 3.1 Repeatability

Analysis of the same image repeated twice by the same user revealed a high value of intra-user repeatability with a repeatability index score of 98.5% and a measurement error of 1.5%, and that by different users also revealed a high value of inter-user repeatability with a repeatability index score of 98.4% and a measurement error of 1.6%.

### 3.2 Wing size variation

**Fig. 4:**
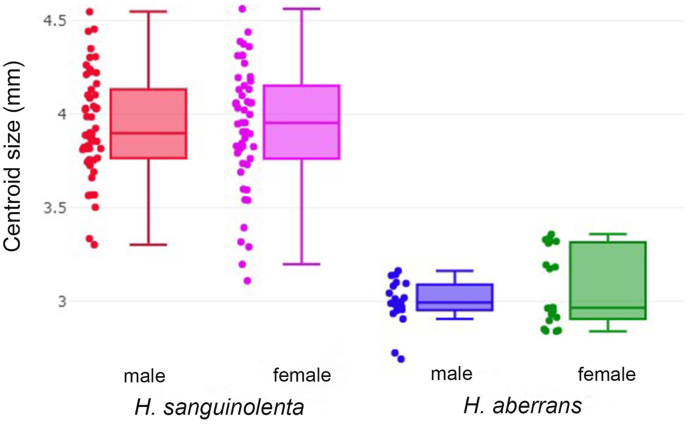
The boxplot of the centroid size variations in male and female *Haematobosca sanguinolenta* and *H. aberrans*. The median dividing the 25th and 75th quartiles is represented by the horizontal line crossing each box.

The centroid size variation in male and female *H. sanguinolenta* and *H. aberrans* is showed by boxplots (Fig. 4). The wing size of male and female *H. sanguinolenta* was 3.94 and 3.93 mm, respectively, and that of male and female *H. aberrans* was 2.99 and 3.07 mm, respectively. *H. sanguinolenta* had a significantly larger wing size than *H. aberrans* in both sexes. The wing size showed no significant difference between male and female flies (Table 2).

**Table 2:** Mean centroid sizes of male and female *H. sanguinolenta* and *H. aberrans*, and statistically significant differences.

Species	Sex	*n*	Mean (mm)	Max-Min	Variance	SD
*H. sanguinolenta*	Male	50	3.94^a^	4.55–3.30	0.08	0.28
	Female	50	3.93^a^	4.56–3.11	0.11	0.33
*H. aberrans*	Male	20	2.99^b^	3.16–2.69	0.01	0.12
	Female	20	3.07^b^	3.36–2.84	0.04	0.20

Statistically significant differences (*p* < 0.05) in wing CS are indicated by different letters.

### 3.3 Wing shape variation

**Fig. 5:**
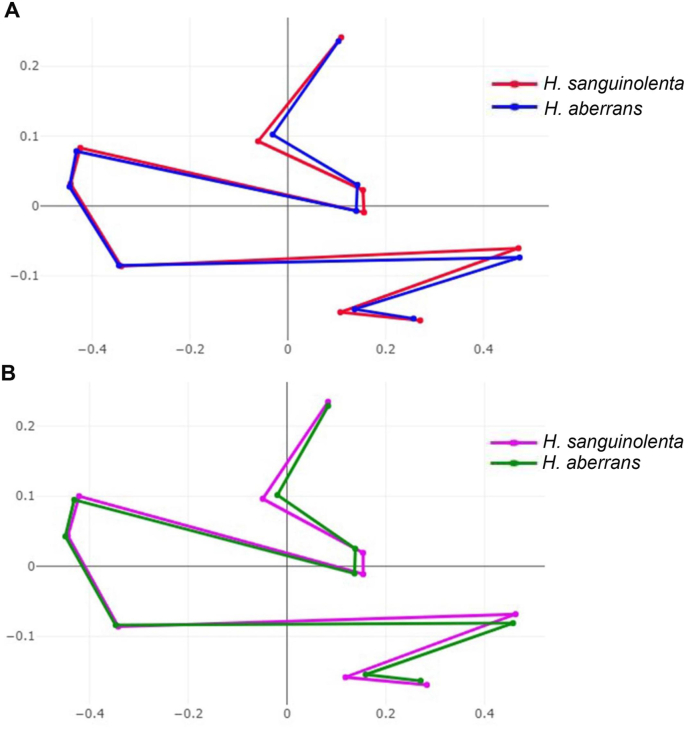
Mean shape of male (A) and female (B) *Haematobosca sanguinolenta* and *H. aberrans* after Procrustes superimposition.

**Fig. 6:**
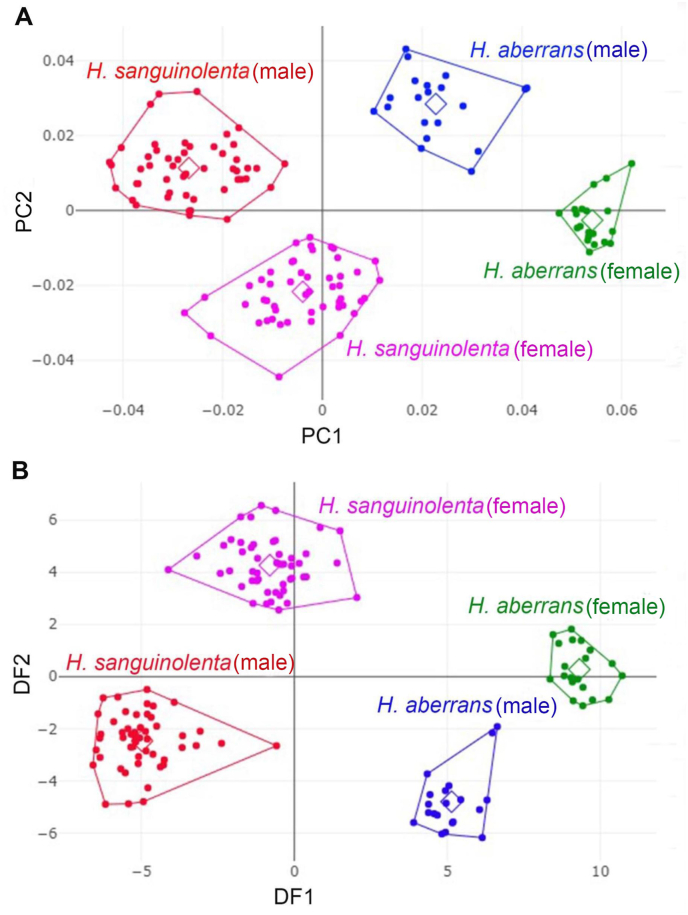
Factor map of the first two principal components (PC1, 47% as horizontal axis and PC2, 33% as vertical axis) of wing shape variables (**A**) and factor map of the first two discriminant factors (DF1, 66.8% as horizontal axis and DF2, 31.8% as vertical axis, the two discriminant factors represent 98.6% of the total discriminant space) of wing shape variables (**B**). Each point represents the individuals of male and female *Haematobosca sanguinolenta* and *H. aberrans*, and each polygon corresponds to a different species and sex. Squares represent the mean values in each group.

The mean shape of the superimposed configurations between male and female *H. sanguinolenta* and *H. aberrans* revealed the most visible landmark displacement in the upper and lower parts of the wing (landmarks 2, 3, 4, 8, 9, and 10) (Fig. 5). The factor map of the first 2 PC as well as the factor map of the first two DFs showed a clear separation between species and sexes (Fig. 6A and B). The pairwise Mahalanobis distances of wing shape showed highly significant differences between species and sexes of the flies (*p* < 0.001), ranging from 7.00 (male and female *H. aberrans*) to 14.57 (male *H. saguinolenta* and female *H. aberrans*) (Table 3).

**Table 3:** Mahalanobis distances of the wing shape for male and female *Haematobosca sanguinolenta* and *H. aberrans*, and *p* values (above diagonal).

Species	*H. saguinolenta* (male)	*H. saguinolenta* (female)	*H. aberrans* (male)	*H. aberrans* (female)
*H. saguinolenta* (male)	–	<0.001	<0.001	<0.001
*H. saguinolenta* (female)	7.96	–	<0.001	<0.001
*H. aberrans* (male)	10.51	10.88	–	<0.001
*H. aberrans* (female)	14.57	10.98	7.00	–

**Fig. 7:**
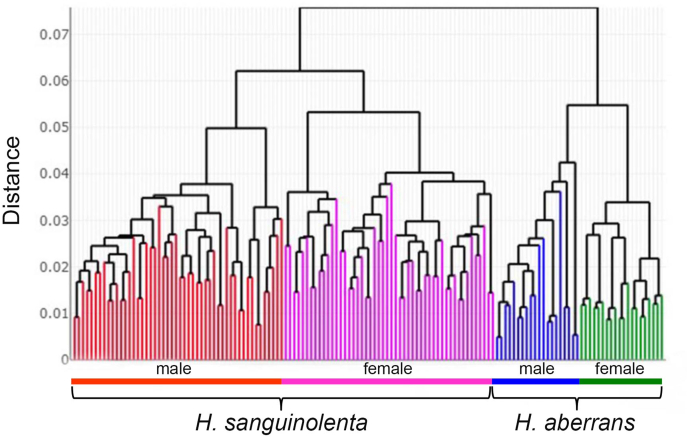
Hierarchical agglomerative clustering tree based on shape similarities of each individual for male and female *Haematobosca sanguinolenta* and *H. aberrans*. Euclidean distances were used for the construction of the tree.

The HAC tree revealed that male and female *H. sanguinolenta* and *H. aberrans* were clearly separated into distinct clusters, and the same species were clustered together (Fig. 7).

### 3.4 Validated classification

The cross-validated classification based on wing size and wing shape for *H. sanguinolenta* and *H. aberrans* yielded overall accuracy scores of 47.1% and 99.3%, respectively. Size-based classification resulted in a relatively low accuracy rating in males (18.6%, 13/70) and a moderate accuracy rating in females (75.7%, 53/70), but the shape-based classification yielded a perfect accuracy rating in both males (98.6%, 69/70) and females (100%, 70/70) (Table 4).

**Table 4:** Percentages of accuracy scores for male and female *Haematobosca sanguinolenta* and *H. aberrans* based on wing size and wing shape using a cross-validated classification.

Species	Sex	Size		Shape	
		% Accuracy	Assigned/Observed	% Accuracy	Assigned/Observed
*H.sanguinolenta*	Male	4	2/50	100	50/50
	Female	82	41/50	100	50/50
*H. aberrans*	Male	55	11/20	95	19/20
	Female	60	12/20	100	20/20
Total		47.1	66/140	99.3	139/140

### 3.5 Allometry

**Fig. 8:**
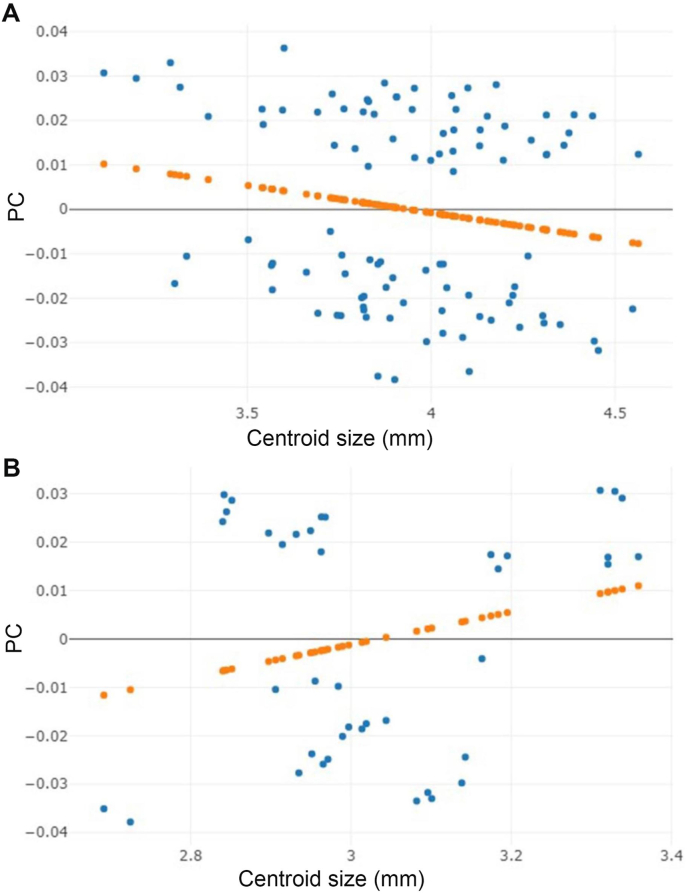
Linear regression between centroid size and the first shape-based principal component (PC) of *Haematobosca sanguinolenta* (A) and *H. aberrans* (B). Linear regression prediction is shown by the orange dots. (For interpretation of the references to colour in this figure legend, the reader is referred to the Web version of this article.)

The allometric effect was very low in both *H. sanguinolenta* (r^2^ = 3) and *H. aberrans* (r^2^ = 5.7), but it was not statistically significant (*p* > 0.05) (Fig. 8). Therefore, the wing size changes of these flies exerted no effect on their wing shape changes.

### 3.6 Identification of test specimens

The 20 additional specimens (10 males and 10 females) were collected from other geographic locations. They served as test specimens for comparison with our study material, which included *H. sanguinolenta* (50 males and 50 females) and *H. aberrans* (20 males and 20 females).

**Fig. 9:**
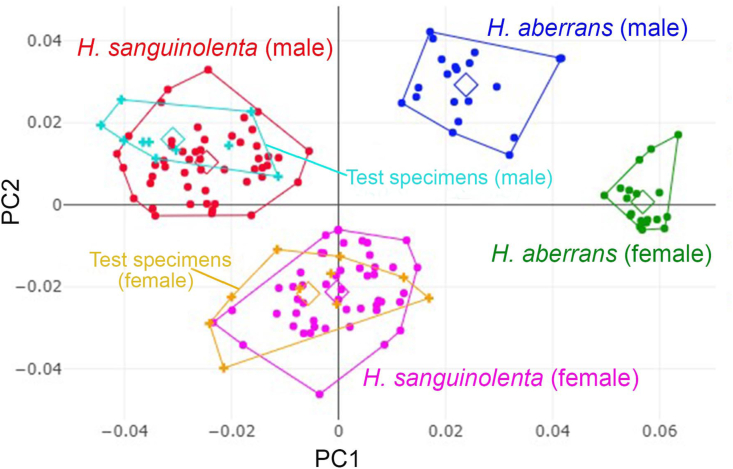
Factor map of the principal components (PC1, 46% as horizontal axis and PC2, 22% as vertical axis) from wing shape variables of test specimens (male and female) and reference data of *Haematobosca sanguinolenta* and *H. aberrans*. Squares represent mean values in each group.

**Fig. 10:**
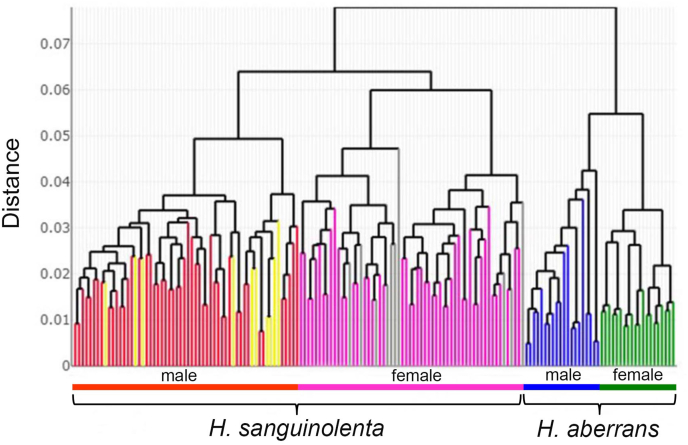
Hierarchical agglomerative clustering tree based on shape similarities of test specimens (male as yellow and female as gray) and reference data of *Haematobosca sanguinolenta* and *H. aberrans*. Euclidean distances were used for the construction of the tree. (For interpretation of the references to colour in this figure legend, the reader is referred to the Web version of this article.)

The total specimen classification, including the reference data, and test specimens, is illustrated by the factor map of the first two PCs (Fig. 9). Each test specimen was correctly assigned to the morphological position corresponding to the reference groups. Moreover, the HAC tree demonstrated that each test specimen was clustered within its groups (Fig. 10).

## 4 Discussion

Our study demonstrated that the two *Haematobosca* flies found in Thailand, viz., *H. sanguinolenta* and *H. aberrans*, can be successfully distinguished, and identified using the landmark-based GM method. Because of their similar morphologies and coexistence in the same habitat (Changbunjong et al., 2020), it can be difficult to distinguish them based solely on physical appearance. In Thailand, *H. sanguinolenta* has been documented in the northern, northeastern, and western regions (Tumrasvin and Shinonaga, 1978; Changbunjong et al., 2012, 2013), whereas *H. aberrans* is still being reported in the northern region of the country (Pont et al., 2020; Changbunjong et al., 2020). Therefore, the specimens used in the GM analysis were obtained only from Chiang Mai Province in northern Thailand, and they were also used as reference data to identify the other specimens collected from other geographic locations. A limitation of our study is the lack of *H. aberrans* for testing as a test specimen because this species is rare compared with *H. sanguinolenta* and could not be collected from the other sites.

The initiative of applying GM to distinguish individuals among stomoxyine fly species was demonstrated by Changbunjong et al. (2016a). They used 10 landmark positions on the wing for distinguishing morphologically similar species of *Stomoxys* flies (*S. pullus*, *S. uruma*, and *S. indicus*). Subsequently, it was evidenced that these landmark positions can be used to discriminate individuals among the other *Stomoxys* flies, namely *S. bengalensis*, *S. calcitrans*, and *S. sitiens* (Changbunjong et al., 2023) and can also be used to explore the phenotypic variation of *S. calcitrans* (Chaiphongpachara et al., 2022b) as well as the sexual dimorphism of *H. aberrans* (Changbunjong et al., 2020). Therefore, we also used the same landmark positions for the GM analysis in the present study.

The GM analysis of wing size variation using mean CS revealed that the wing size of *H. sanguinolenta* was significantly larger than that of *H. aberrans* in both sexes. Our results indicate that the wing size could help distinguish between *H. sanguinolenta* and *H. aberrans.* However, based on the cross-validated classification, the wing size showed only a moderate accuracy in female flies and a poor accuracy in male flies. Hence, we do not suggest using the wing size to distinguish these flies if the sex of the flies is unknown. In fact, it has also been shown that the wing size is unsuitable for the differentiation of insect species because it is frequently influenced by environmental factors (Jirakanjanakit et al., 2007; Morales Vargas et al., 2010; Baleba et al., 2019; Phanitchat et al., 2019; Chaiphongpachara et al., 2022b; Chaiphongpachara et al., 2022c).

Based on the wing shape analysis, GM was highly effective in distinguishing *Haematobosca* species in Thailand, which is supported by the mean shape configurations (Fig. 5), morphospace (Fig. 6A), discriminant space (Fig. 6B), and the HAC tree (Fig. 7) as well as a very high overall accuracy score (99.3%). Although the results of the morphospace, discriminant space, and HAC tree demonstrated excellent group separation, the cross-validated classification did not show 100% accuracy of the correct classification because one specimen of male *H. aberrans* was classified as female of its species. The difference in wing shape between *H. sanguinolenta* and *H. aberrans* was entirely consistent with the *COI* sequences for species separation (Changbunjong et al., 2020). Additionally, it was also compatible with the morphological classification where *H. sanguinolenta* could be clearly distinguished from *H. aberrans* by the presence of an anterior katepisternal seta. The results of the allometric effect analysis also indicated that the wing shape variation of *H. sanguinolenta* and *H. aberrans* was not affected by the wing size variation. According to our results, genetic divergence is probably responsible for the difference in wing shape between *Haematobosca* species in Thailand.

In this study, we also determined the sexual shape dimorphism in the wing of *H. sanguinolenta* and *H. aberrans*, indicating that the distinction between the males and females of these flies is based on wing shape variation. Our findings confirmed the sexual shape dimorphism in the wing of *H. aberrans* that was previously established using specimens from the same population (Changbunjong et al., 2020). In general, the sexual shape dimorphism of wings frequently exerts a passive effect on the sexual size dimorphism (or allometry) (Ruangsittichai et al., 2011); however, this was not the case in the present study (non-allometric effect). Nonetheless, non-allometric effects such as flight behavior and mating systems can be related to wing shape variations (Gidaszewski et al., 2009).

The results obtained using our study material or reference data to identify test specimens collected from other geographical locations were satisfactory. All test specimens were correctly classified according to their species and sexes, as shown in Fig. 9, Fig. 10. This increased our confidence in their potential application as reference data for further identification of the unknown *Haematobosca* specimen in our country. The use of GM material as a public reference for species identification has been demonstrated in various insects, such as horse flies (Changbunjong et al., 2021), *Stomoxys* flies (Changbunjong et al., 2023), and ants (Samung et al., 2022). However, measurement errors are an essential factor that must be considered for its implementation (Changbunjong et al., 2021). In the present study, we demonstrated that digitizing the wings of *H. sanguinolenta* and *H. aberrans* yielded excellent precision, irrespective of the same user (98.5% intra-user repeatability) or two different users (98.4% inter-user repeatability), so that our material can be utilized as reference data for the identification of these two species. Finally, we propose that wing GM can be used as a supplement to conventional morphology identification, particularly for specimens that have been damaged or have lost their katepisternal seta due to specimen collection and processing in the field.

## 5 Conclusions

Our study investigated the effectiveness of landmark-based GM of the wing to distinguish and identify two *Haematobosca* flies, namely, *H. sanguinolenta* and *H. aberrans*, in Thailand. The results showed that GM was highly effective in distinguishing between these fly species based on the wing shape. Moreover, the material obtained from this study served as reference data in the species identification of specimens collected from other geographic locations. Therefore, the GM method can be used in addition to morphological identification when a specimen of fly is damaged or when there is a loss of crucial distinguishing characteristics. In future research, it is necessary to examine the morphological variation in the wing morphometry of *Haematobosca* populations in terms of phenotypic plasticity as well as population genetic variation in each region of Thailand, which is essential for developing effective population control strategies.

## Credit author statement

**Nusara Ardkhongharn:** Investigation, Validation, Writing—original draft preparation. **Romyakorn Ravichotikul:** Investigation, Validation, Writing—original draft preparation. **Patthanan Aksornchai:** Investigation, Validation, Writing—original draft preparation. **Thekhawet Weluwanarak:** Investigation. **Tanawat Chaiphongpachara:** Methodology, Validation, Investigation, Writing—review and editing. **Tanasak Changbunjong:** Conceptualization, Methodology, Validation, Investigation, Resources, Data curation, Writing—original draft preparation, Writing—review and editing, Project administration, Funding acquisition.

## Data availability statement

The wing images used in this study are openly available in FigShare at doi https://figshare.com/articles/figure/Haematobosca_spp_images_for_Geometric_Morphometrics/22658929.

## Declaration of competing interest

The authors declare that they have no known competing financial interests or personal relationships that could have appeared to influence the work reported in this paper.
